# Perceived Autonomy and Anticipated Turnover: The Mediation Role of Burnout Among Critical Care Nurses

**DOI:** 10.3390/healthcare13060652

**Published:** 2025-03-17

**Authors:** Rawaih Falatah, Eqbal Alfi

**Affiliations:** 1 Nursing Administration and Education Department, College of Nursing, King Saud University, Riyadh 11362, Saudi Arabia; 2 937 Operation Department at Makkah Healthcare Cluster, Executive Administration at Patient Experience, Executive Administration at Institutional Excellence and Quality, Makkah 24231, Saudi Arabia; ialfi@moh.gov.sa

**Keywords:** autonomy, burnout, emotional exhaustion, anticipated turnover, critical care, nurses

## Abstract

**Background:** Several factors are associated with the nurse anticipated turnover rate, including factors related to the staff, organisation, and management and leadership practices. Nurses in critical care areas (intensive care units and emergency rooms) demonstrated a higher anticipated turnover rate compared to nurses in other healthcare areas. There is evidence that imposing autonomy through managerial intervention is imperative, leading to increased confidence among nurses in terms of decision-making and enhancing their well-being, thus improving their performance, job satisfaction, and job retention. **Aim:** The aim of this study was to examine the mediating effect of burnout on the association between perceived autonomy and anticipated turnover among critical care nurses. **Design:** We utilized a cross-sectional descriptive survey design. **Methods:** We collected data from 134 nurses working in critical care areas using convenient sampling through an online survey. For data analysis, we utilized the Hayes process macro to examine the mediation model. **Results**: In this study, perceived autonomy was a significant negative predictor of nurses’ anticipated turnover, and this association is partially mediated by nurses’ emotional exhaustion. **Conclusions:** Nurse managers and policymakers should strive to support the autonomy and psychological well-being of critical care nurses as they play an important professional role within the healthcare team.

## 1. Introduction

Anticipated turnover is used interchangeably with turnover intention, intention to leave, and intention to quit; and is defined as an employee’s intention to leave their current workplace to pursue another employment in the near future [1,2]. Turnover leads to increased staff shortage, which negatively impacts care outcomes as well as organisational and staff outcomes [3]. In critical care, nurse shortage has been ongoing for decades [4]. Among the reported predictors of turnover intention are nurses’ sociodemographic characteristics, job satisfaction, job commitment, workload, and bullying [5,6]. According to the literature, nurse turnover is usually measured using turnover intention or anticipated turnover and it ranges between 14.06% [7] and 94% [8]. In the critical care area, Khan, Jackson, Stayt, and Walthall [9] reported nurse turnover intention to be between 17% and 48.1%. In a review of nurse turnover in Saudi Arabia literature, the highest reported rate was 60%, while the lowest was 17% [10]. Indeed, the International Council of Nursing considered nursing turnover a global issue and urged governments to take actions to mitigate the risk of increased turnover [11].

These high anticipated turnover rates are linked in the literature with high levels of burnout among nurses [12], which is one of the most common psychological problems among nurses worldwide [13,14]. Specifically, the problem of nurse burnout is higher in critical care areas [15]. In the literature, burnout is defined and measured by three components: emotional exhaustion, depersonalisation, and personal accomplishment [16]. According to a systematic review and meta-analysis of 113 studies, including 45,539 nurses from 49 countries, the prevalence of nurse burnout worldwide is approximately 1 in 10 nurses [14]. Among critical care nurses, one-third (33.9%) met the criteria for burnout [15]. Furthermore, among a sample of nurses in Saudi Arabia, nurses working in critical care units had a higher percentage of burnout compared to nurses working in general wards. The contributing factors for nurse burnout include night shifts, a disproportional nurse-to-patient ratio, the presence of critical and complex cases that require careful attention, and high mortality rates [17,18]. On the other hand, factors such as job satisfaction and autonomy are associated with lower burnout among nurses [19].

Nurse autonomy is important in the nursing practice to ensure the quality and safety of care [20,21,22]. Generally, autonomy is defined as the ability to make and act on decisions. In the context of nursing, autonomy refers to the ability to engage in activities within the scope of the nursing practice without seeking permission from others [20]. In a study conducted among nurses in the United Kingdom to assess their perception of autonomy, six themes were identified: working independently, working in a team, having professional skills and knowledge, involvement in autonomy, boundaries around autonomy, and developing autonomy requiring support [23]. As the last theme in Oshodi’s study suggests, the development of autonomy among staff nurses requires support. Nurse managers are well-positioned to support staff nurses and promote autonomy among them [24].

Three dimensions of nurse autonomy have been identified: clinical autonomy, job autonomy, and control over practicing nursing [25]. Numerous studies have reported moderate nurse autonomy, including a study of critical care nurses [26]. Nonetheless, nurses associate autonomy with their clinical practice and the specific work environment of their ward rather than with a broader professional framework [2,18]. Among the identified predictors of nurse autonomy are age, experience [20], gender, area of practice [27], education, hospital bed capacity [19], and supervisor support [25].

The outcomes of staff nurses’ autonomy were explored in nursing literature. Enns, Currie, and Wang [28] found that promoting autonomy in the work environment reduces the rate of depression and absenteeism among nurses. In a surgical unit, nurse autonomy was positively associated with a reduction in 30-day mortality and failure to rescue [22]. Moreover, imposing autonomy through managerial intervention is imperative because it leads to increased confidence among nurses in terms of decision-making and enhances their performance, job satisfaction, job commitment, and retention [19], especially in critical care units where nurses interact with critically ill patients, which increases the incidence of burnout and turnover [27]. Nonetheless, one study revealed a positive association between nurse autonomy and moral distress [29].

Despite the importance of nurse autonomy in improving staff satisfaction and retention, the impact of the staff nurses’ perceptions of nurse managers’ actions to promote nurse autonomy on staff nurse burnout has not been explored. Additionally, the mediation impact of burnout components on the association between staff nurses’ perceptions of nurse managers’ actions to promote nurse autonomy on staff nurses’ anticipated turnover has not been examined. Thus, our study aims to explore the association between nurse managers’ actions to promote nurse autonomy, burnout, and anticipated turnover.

### Study Aim and Questions

This study aimed to examine the mediating effect of burnout components on the association between the nurse perception of nurse managers’ actions to promote nurse autonomy and anticipated turnover among critical care nurses. The following questions were addressed in this study:

1. What is the association between staff nurses’ perceptions of nurse managers’ actions to promote autonomy, burnout components, and anticipated turnover?
2. Do burnout components mediate the association between staff nurses’ perceptions of nurse managers’ actions to promote autonomy and anticipated turnover?

## 2. Materials and Methods

### 2.1. Design

We used a cross-sectional descriptive survey design to test the mediation effect of burnout components on the association between staff nurses’ perceptions of nurse managers’ actions to promote autonomy and anticipated turnover among critical care nurses.

#### 2.1.1. Study Setting and Sample

The study was conducted in the critical care area of two hospitals in the western region of Saudi Arabia. The first hospital has around 350 bed capacity and 1469 nurses. The second hospital has a bed capacity of 72 beds and 116 nurses. Both hospitals were selected because they are specialized hospitals that have critical care units. A convenience sample of nurses was invited to participate in the study. The required sample size was calculated using G*Power Version 3.1.9.6 [30]. The effect size, α, and power were 0.3, 0.05, and 0.95, respectively; the sample size was estimated as 134 participants. The medium effect size of 0.3 was selected as a conservative estimation to ensure adequate statistical power [31]. The inclusion criteria for the study were staff nurses of any nationality and gender who were working in critical care areas for six months or more. Nurses who were not working in critical care areas, nursing students, and nursing interns were excluded. In addition, we excluded nurses with management assignments. The survey link was sent to 150 nurses, and 100 of them participated within two weeks. Reminder emails were sent on the third week and the sixth week. The total number of participants was 134, which resulted in a response rate of 89.3%.

#### 2.1.2. Measurements

The study questionnaire was composed of four parts: sociodemographic questions such as age, gender, nationality, qualification, working area, and years of experience; the Nurse Managers’ Actions (NMAs) Scale to promote nurse autonomy; the Maslach Burnout Inventory Human Services Survey for Medical Personnel (MBI-HSS (MP)); and the Anticipated Turnover Scale (ATS).

##### The Nurse Managers’ Actions (NMAs) to Promote Nurse Autonomy Scale

The second part of the questionnaire was the NMAs to promote nurses’ autonomy scale, which was developed by Mrayyan [30]. This scale was used to ask nurses how often the nurse manager performed certain actions, such as “supports nurses to resolve conflicts with physicians, patients, and colleagues” and “supports staff nurses’ autonomous decision-making”. It is an 11-item 5-point Likert-type scale: 1 = never, 2 = seldom, 3 = sometimes, 4 = usually, 5 = always. An exploratory factor analysis (EFA) was carried out on the scale, resulting in a three-factor structure that explained 66.5% of the total variance. The factors identified were support for autonomous decision-making, promotion of professional development, and facilitation of participative management [32,33]. The scores on the scale range from 11 to 55. In the current study, the scale had a Cronbach’s α = 0.94. Al-Hamdan, Bawadi, Bawadi, and Mrayyan [34] reported the same Cronbach’s α.

##### The Maslach Burnout Inventory (MBI)

The third part of the questionnaire was the MBI-HSS (MP) [16]. It contains twenty-two items, including three subscales: the emotional exhaustion (EE) subscale (nine items) to measure the extent of feeling emotional and being exhausted; the depersonalisation (DP) subscale (five items) to assess negative feelings toward recipients of care; and personal accomplishment (PA) subscale (eight items) to assess the feeling of success and accomplishment at work. The three subscales were established as a result of factor analysis and have been reported repeatedly in the literature [35]. The MBI-HSS (MP) is scored on a 7-point scale as follows: 0 = never, 1 = a few times a year or less, 2 = once a month or less, 3 = a few times a month, 4 = once a week, 5 = a few times a week, and 6 = every day. The score calculation is performed by two methods: the first, by summing up the survey responses on each subscale to obtain the total score for each of the three subscales, and the second, by dividing the total score of each subscale by the number of each subscale item to obtain three average score variables. The average score is usually reported; however, the total score is important to determine the sample’s burnout level (high, moderate, and low) for each subscale. High emotional exhaustion and depersonalization scores and low personal accomplishment scores indicated a high level of burnout. Reliability, estimated by Cronbach’s α, was α = 0.90 for the emotional exhaustion subscale, α = 0.79 for the depersonalization subscale, and α = 0.71 for the personal accomplishment subscale [16]. In the current study, emotional exhaustion was α = 0.877, depersonalization was α = 0.672, and personal accomplishment was α = 0.754.

##### Anticipated Turnover Scale (ATS)

The fourth part of the questionnaire was the Anticipated Turnover Scale (ATS), developed by Hinshaw and Atwood [36], to assess the possibility of nurses quitting their jobs. It consists of 12 items rated on a 7-point Likert-type scale (7–1 for positive items and 1–7 for negative items). The total score, which ranges from 12 to 84, is the sum of all items in the scale divided by the total number of items. The original validity test of the scale yielded a unidimensional scale, focusing on a single factor representing anticipated turnover intentions. According to Hinshaw and Atwood [36], the internal consistency reliability estimated using Cronbach’s α was 0.84 [36], and for the current study, it was α = 0.717.

#### 2.1.3. Data Collection Procedure

An online survey was formulated using Google Forms in Arabic and English. We sent IRB approval with a researcher support letter through email to the directors of the two hospitals to facilitate our work. After obtaining the directors’ approval, we sent emails to the education and training departments of the two hospitals and explained the project, its purpose, and the inclusion criteria with the survey link attached. The education and training departments asked critical care department head nurses to forward the survey link to staff nurses through email and WhatsApp (version 25.6.73) groups. Participants provided informed consent at the beginning of the survey. Two follow-up emails were sent four weeks apart from the initial email. Data were collected between 20 December 2020 and 8 March 2021.

#### 2.1.4. Ethical Considerations

IRB approval was obtained before data collection from the King Saud University College of Medicine Committee IRB No. (E-20-4717). Hospital permissions were obtained before collecting any data. The staff nurses were informed that their participation was voluntary and their information would be kept anonymous. The data were stored in a password-protected drive that could only be accessed by the research team.

#### 2.1.5. Data Analysis

The Statistical Package for Social Science (SPSS) version 28 was used to analyse the data. Initially, data were cleaned, and the missing data were managed using multiple imputations. Univariate analysis was used to describe the sample and explore violations of the statistical assumptions (normality test, as shown in Table 1). Bivariate analysis was used to examine the first study question using correlation. Multivariate analysis was used to examine the second question (the mediation analysis), using the Hayes process macro for SPSS [37].

## 3. Results

### 3.1. Demographic Data of the Participants

A total of 134 nurses participated in this study. The average age of the study participants was 34.13 (SD = 6.45) years, and the range was 32 years. The results showed that most of the participants (92.5%) were female and 74.6% had a bachelor’s degree. The average years of experience in critical care area was 7.60 (SD = 5.09) years, and the range was 20 years. The majority (71.6%) of participants were non-Saudi. Additionally, the distribution of participants in the critical care units was as follows: dialysis units (35.1%), cardiac care units (23.9%), emergency rooms (21.6%), and general adult critical care units (18.7%).

### 3.2. Study Variables

The descriptive statistics of the study variables are presented in Table 1. The mean of the NMA scale to promote nurses’ autonomy was 3.320 (SD = 0.898, range = 3.82), which indicates that nurses perceived that their managers sometimes took actions to promote nurse autonomy. For the mean of the MBI-HSS (MP) subscales, the highest was for personal accomplishment ($\bar{x}$ = 4.811, SD = 1.035, range = 5.63), followed by emotional exhaustion ($\bar{x}$ = 2.699, SD = 1.476, range = 5.78), and depersonalisation ($\bar{x}$ = 1.311, SD = 1.243, range = 6). Finally, the mean of the ATS was 3.675 (SD = 0.925, rang = 5.17).

Table 2 shows the prevalence of burnout among the nurses. A total of 42.4% of the sample had high emotional exhaustion, and 26.9% reported feelings of high depersonalisation. Additionally, 15.7% reported low personal accomplishments. High emotional exhaustion, depersonalisation, and low personal accomplishment scores indicated a high level of burnout. The results revealed that while a high percentage of the sample reported high levels of emotional exhaustion, a high percentage reported low depersonalisation and high personal accomplishment. Thus, it cannot be concluded that this sample of critical care nurses has experienced burnout. Nonetheless, it can be concluded that they have high levels of emotional exhaustion.

A series of chi-square tests were performed to explore the relationships between two independent variables (IVs), gender and nationality, and five dependent variables (DVs), NMAs, EE, DP, PA, and ATS. The findings are presented in Table 3. For gender, notable associations were identified with NMAs, where χ^2^(40) = 64.53, *p* = 0.008 and Cramér’s V = 0.694; EE, where χ^2^(43) = 78.73, *p* < 0.001 and Cramér’s V = 0.767; DP, where χ^2^(24) = 60.99, *p* < 0.001 and Cramér’s V = 0.675; and ATS, where χ^2^(41) = 82.11, *p* < 0.001 and Cramér’s V = 0.783. No notable link was found between gender and PA, where χ^2^(29) = 21.74, *p* = 0.831 and Cramér’s V = 0.403.

For nationality, notable correlations were identified with NMAs, where χ^2^(40) = 56.85, *p* = 0.041 and Cramér’s V = 0.651; PA, where χ^2^(29) = 46.45, *p* = 0.021 and Cramér’s V = 0.589; and ATS, where χ^2^(41) = 73.50, *p* = 0.001 and Cramér’s V = 0.741. Nonetheless, no notable correlations were identified between nationality and EE, where χ^2^(43) = 47.00, *p* = 0.312 and Cramér’s V = 0.592, or DP, where χ^2^(24) = 32.53, *p* = 0.114 and Cramér’s V = 0.403. Effect sizes (Cramér’s V) ranged from moderate to large, suggesting that the strength of the relationships differed based on the specific IV-DV combination.

Pearson’s and Spearman’s correlation coefficients were computed to examine the relationship between sociodemographic characteristics (age, years of experience, and qualification) and the study factors (NMAs, EE, DP, PA, and ATS). Spearman’s correlation was applied for qualification because of its ordinal characteristics, whereas Pearson’s correlation was utilized for the remaining variables. The findings are shown in Table 4.

Age did not exhibit a significant correlation with any of the study variables, although it displayed a nonsignificant negative correlation with ATS (r = −0.163, *p* > 0.05). Likewise, years of experience showed no significant correlations with NMAs, EE, DP, PA, or ATS. Regarding qualification, no significant correlations were found with NMAs (ρ = 0.037, *p* > 0.05), EE (ρ = 0.085, *p* > 0.05), PA (ρ = 0.092, *p* > 0.05), ATS (ρ = −0.141, *p* > 0.05) or DP (ρ = 0.165, *p* > 0.05).

Among the study variables, NMAs demonstrated a significant negative relationship with ATS (r = −0.243, *p* < 0.01). EE showed a significant negative correlation with NMAs (r = −0.205, *p* < 0.05) and a positive correlation with ATS (r = 0.247, *p* < 0.01). DP showed a positive correlation with both NMAs (r = 0.167, *p* > 0.05) and EE (r = 0.464, *p* < 0.01), whereas PA did not display significant correlations with any other variables. These results emphasize important associations among NMAs, EE, ATS, and sociodemographic variables, with several correlations achieving statistical significance.

Research Question 1: What is the association between staff nurses’ perceptions of nurse managers’ actions to promote autonomy, burnout components, and anticipated turnover among staff nurses?

Nurses’ perception of managers’ actions to promote nurse autonomy was significantly and negatively associated with the nurses’ emotional exhaustion (r = −0.205, *p* = 0.017) and anticipated turnover (r = −0.243, *p* = 0.005). In addition, emotional exhaustion was significantly and positively associated with depersonalisation (r = 0.464, *p* < 0.001) and anticipated turnover (r = 0.247, *p* = 0.004).

Research Question 2: Do nurses’ burnout components mediate the association between staff nurses’ perceptions of nurse managers’ actions to promote autonomy and anticipated turnover among staff nurses?

**Figure 1:**
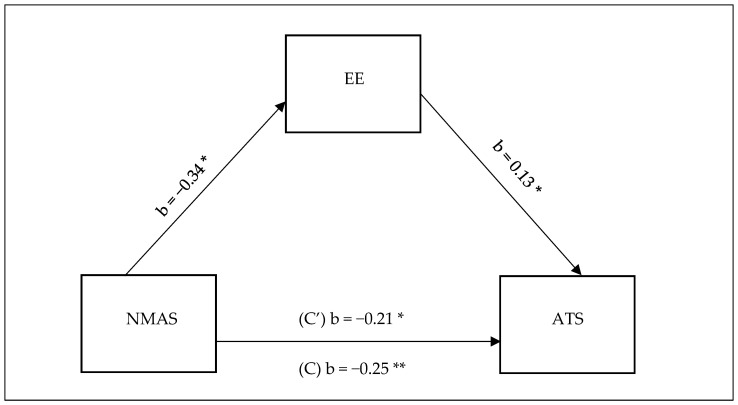
Model for the association between staff nurses’ perceptions of nurse managers’ actions to promote autonomy on anticipated turnover. * *p* < 0.05, ** *p* < 0.01.

Given that both depersonalisation and personal achievement had no statistically significant associations with staff nurses’ perception of nurse managers’ actions to promote nurse autonomy and anticipated turnover, we performed mediation model analysis using Hayes PROCESS Model 4 with one mediator. The purpose was to examine the mediation effect of emotional exhaustion on the association between staff nurse perceptions of nurse managers’ actions to promote autonomy and anticipated turnover among critical care nurses (Table 5, Figure 1). Overall, the model was statistically significant and explained 4.21% of the change in nurses anticipated turnover [F (5.80, 132) = 3.39, *p* = 0.017]. The results revealed a significant indirect effect of staff nurse perceptions of nurse managers’ actions to promote nurse autonomy on anticipated turnover (b = −0.044, t = −2.368). Furthermore, the direct effect of staff nurses’ perceptions of nurse managers’ actions to promote nurse autonomy on anticipated turnover in the presence of the mediator was also found significant (b = −0.206, *p* = 0.019). Thus, it can be concluded that emotional exhaustion partially mediated the relationship between nurse managers’ actions to promote nurse autonomy and anticipated turnover. The mediation analysis summary is presented in Table 5 and Figure 1 is a visual representation of the relationship.

## 4. Discussion

To the best of our knowledge, this is the first study to examine the mediating effect of burnout components on the association between staff nurses’ perceptions of nurse managers’ actions to promote autonomy and anticipated turnover among critical care nurses. The participants thought that sometimes their nurse managers took action to promote their autonomy. This result is comparable to the results reported by Al-Hamdan et al. [34] and Labrague et al. [19]; however, Bakeer and Nassar (2018) [24] stated that approximately 69% of a sample of nurses from Menofia Governorate, Egypt, reported feeling that their nurse managers perform many actions to promote their autonomy. Moreover, Maharmeh [27] revealed that a sample of Jordanian critical care nurses reported being autonomous in both decision-making and decision participation in their clinical areas. The perception of the nurses in the current study regarding nurse managers’ actions to promote nurse autonomy might be related to the state of the nursing profession in Saudi Arabia. Although there is gargantuan governmental support to the nursing profession, an important component of the profession is missing, which is the scope of practice [38]. It can be argued that without a clear scope of practice, nurses can hardly identify their professional, ethical, and legal boundaries, which might negatively impact nurses’ autonomy. It could be further argued that the lack of nursing scope of practice and its impact on nurses autonomy might amplify negative nursing workforce outcomes such as decreased satisfaction and commitment and increased turnover [20].

As nurse turnover persists as a challenge for healthcare systems worldwide, our sample of critical care nurses showed moderate anticipated turnover. This finding is comparable to that of Albougami et al.; Irshad, Khattak, Hassan, Majeed, and Bashir; and Khattak, Saeed, Rehman, and Fayaz [39,40] who reported moderate turnover intention. However, studies such as those of Labrague and de los Santos [19] and Qi, Wei, Li, Liu, and Xu [41] reported low turnover intention among Philippine and Chinese nurses. This lower-than-average turnover intention might be attributed to the relative abundance of nursing supplies compared to that in other countries, including Saudi Arabia, which might moderately restrict the mobility of the nursing workforce between organisations. Also, the low turnover rates in these countries might be related to the inability to change facilities as the nurses in many cases will be contracted to a specific hospital and not have the option to transfer. Indeed, alternative employment opportunity has been identified as a contributing factor for nurse turnover in Saudi Arabia [42]. The current study added an original contribution to the predictors of anticipated turnover in Saudi Arabia. The study has identified nurses’ perception about the nurse managers’ action to promote nurse autonomy as a significant predictor of nurses anticipated turnover.

In the literature, burnout has also been found to be a contributing factor to nurses turnover intentions. Although we found that our sample of critical care unit nurses did not report burnout, they reported high emotional exhaustion. Jalili, Niroomand, Hadavand, Zeinali, and Fotouhi [43] revealed high burnout among a sample of Iranian nurses. Both the studies, Jalili et al., and our study, were conducted during the COVID-19 pandemic; however, the level of burnout among the two samples was contradictory with our study reporting lower burnout levels. This may be because of two important factors. First, the pandemic prevailed in the country given that Iran has more cases than Saudi Arabia. Second, the availability of resources, supplies, technology, and support might play a role in lowering burnout among critical care nurses in Saudi Arabia.

Related to our study questions, the findings revealed a statistically significant negative association between nurse managers’ actions to promote nurse autonomy, emotional exhaustion, and anticipated turnover. Additionally, emotional exhaustion was significantly positively associated with depersonalisation and anticipated turnover. Similar findings have been reported by Labrague et al. [19] and Shohani et al. [20]. It could be argued that when critical care nurses perceive themselves as autonomous in the provision of their clinical practice and its related decision-making, they will have higher psychological well-being and it might contribute to decreasing emotional exhaustion and anticipated turnover. Reducing these negative nursing workforce outcomes especially in the critical care setting is thought to improve patients’ outcomes [22].

To the best of our knowledge, this study makes a unique contribution in revaluing nurse managers’ actions to promote nurse autonomy directly and indirectly through burnout, significantly predicting critical care nurses’ anticipated turnover. This finding adds to the limited body of knowledge that attempts to gain a better understanding of nurse turnover. Future studies in this area are needed to build on the current study findings on the impact of critical care nurses’ perception of nurse managers’ actions to promote nurse autonomy on the nursing workforce outcomes. We also recommend the expansion of future studies to include the impact of nurses’ autonomy on patient care outcomes.

### 4.1. Limitations

The findings of our study have two limitations. First, the study was conducted in two hospitals in one Saudi region, which may limit the generalizability of the study findings. Future studies should include more hospitals across the country. Second, this study utilised a cross-sectional design and convenience sample. Both these factors may have impacted the generalizability of the study findings. Future studies should implement an experimental design in which nurse managers’ actions to promote nurse autonomy are manipulated to observe their impact on both nurses and care outcomes.

### 4.2. Recommendations for Practice

The high pace and demanding critical care environment highlight the need for independent, autonomous professional groups that are supported and empowered to be active healthcare team participants. Thus, nurse managers should take action to promote the staff nurses’ autonomy. Policymakers and healthcare nurses should utilise the findings of the current study to develop policies and guidelines that authorise nurse managers to take action to promote the staff nurses’ autonomy. Furthermore, healthcare organisations are recommended to regularly measure nurse autonomy, particularly in critical care units, and take measures to improve nurses’ autonomy through policy, rules, education, and training. Nurses’ professional autonomy should be publicly supported and communicated to all members of the healthcare team. Additionally, nurses’ participation in decision-making through shared governance and organisational-level committees must be supported.

## 5. Conclusions

The current study’s findings indicate that nurse managers’ actions to promote nurse autonomy directly and indirectly predict critical care nurses’ anticipated turnover. Although the sample did not report burnout, they reported high emotional exhaustion and moderate anticipated turnover. The findings suggest that as staff nurses’ perceptions of nurse managers’ actions to promote nurse autonomy increase, nurse burnout decreases, which agrees with previous studies. These findings add to the literature that burnout partially mediates the association between nurse managers’ actions to promote nurse autonomy and nurse anticipated turnover. These findings build on to the theories that link nurses’ well-being with their anticipated turnover and add the role of NMAs to promote nurses’ autonomy in predicting nurses’ anticipated turnover through burnout. The practical implication of this study can be through policy development to empower nurse managers and training them on methods to enhance nurses’ autonomy. Further research is required to replicate the current study and expand it to other nursing areas.

## Figures and Tables

**Table 1:** Descriptive statistics of the study variables (n = 134).

Variables	Mean	SD	Range	Skewness		Kurtosis	
				Statistic	Std Error	Statistic	Std Error
NMAs	3.320	0.898	3.82	−0.057	0.209	−0.531	0.416
MBI-HSS (MP)—EE average	2.699	1.476	5.78	0.312	0.209	−0.891	0.416
MBI-HSS (MP)—DP average	1.311	1.243	6.00	1.334	0.209	1.769	0.416
MBI-HSS (MP)—PA average	4.811	1.035	5.63	−1.556	0.209	2.616	0.416
MBI-HSS (MP)—EE total	24.30	13.29	52.00	0.31	0.209	−0.891	0.416
MBI-HSS (MP)—DP total	6.56	6.22	30.00	1.33	0.209	1.77	0.416
MBI-HSS (MP)—PA total	38.49	8.28	45.00	−1.56	0.209	2.62	0.416
ATS	3.675	0.925	5.17	−0.256	0.209	0.359	0.416

**Table 2:** The study sample classification on MBI-HSS (MP) subscales.

	Subscale	Emotional Exhaustion	Depersonalization	Personal Accomplishment
Fe. (%) Level				
High		**57 (42.4)**	**36 (26.9)**	87 (64.9)
Moderate		21(15.7)	25 (18.7)	26 (19.4)
Low		56 (41.8)	73 (54.5)	**21 (15.7)**

EE (≥27 = high; 19–26 = moderate; 0–18 = low). DP (≥10 = high; 6–9 = moderate; 0–5 = low). PA (≥40 = high; 34–39 = moderate; 0–33 = low). High EE and DP, and low personal Accomplishment indicate burnout.

**Table 3:** Chi-square test for gender (male, female) and nationality (Saudi, Non-Saudi) with the study variables.

Independent Variable (IV)	Dependent Variable (DV)	χ^2^ (df)	*p*-Value	Effect Size (Cramér’s V)
Gender	NMAs	64.527 (40)	0.008	0.694
	EE	78.732 (43)	<0.001	0.767
	DP	60.993 (24)	<0.001	0.675
	PA	21.739 (29)	0.831	0.403
	ATS	82.111 (41)	<0.001	0.783
Nationality	NMAs	56.851 (40)	0.041	0.651
	EE	46.995 (43)	0.312	0.592
	DP	32.530 (24)	0.114	0.403
	PA	46.454 (29)	0.021	0.589
	ATS	73.495 (41)	0.001	0.741

(NMAs, nurse managers’ actions to promote autonomy; ATS, anticipated turnover scale; EE, emotional exhaustion; DP, depersonalisation; PA, personal accomplishment).

**Table 4:** Correlation between the sociodemographic and the study variables.

Variables	1	2	3	4	5	6	7
1. Age							
2. Years of experience	0.656 **						
3. Qualification †	−0.055	0.661 **					
4. NMAs	0.020	−0.019	0.037				
5. EE	0.025	−0.023	0.085	−0.205 *			
6. DP	−0.096	−0.130	0.165	0.167	0.464 **		
7. PA	0.099	0.128	0.092	0.028	0.128	0.018	
8. ATS	−0.163	−0.045	−0.141	−0.243 **	0.247 **	0.091	0.073

(NMAs, nurse managers’ actions; ATS, anticipated turnover scale; EE, emotional exhaustion; DP, depersonalisation; PA, personal accomplishment). † Spearman’s correlation. * *p* < 0.05, ** *p* < 0.01, (2-tailed).

**Table 5:** Mediation analysis summary.

Relationship	Total Effect	Direct Effect	Indirect Effect	Confidence Interval		t-Statistics	Conclusion
NMAS -> EE -> ATS	−0.250 (0.004)	−0.206 (0.019)	−0.044	Lower Bound	Upper Bound	−2.368	Partial Complementary Mediation
				−0.104	−0.003		

## Data Availability

The data presented in this study are available on request from the corresponding author.

## Abbreviations

The following abbreviations are used in this manuscript:

NMAs	Nurse Managers’ Actions
MBI-HSS (MP)	Maslach Burnout Inventory Human Services Survey for Medical Personnel
ATS	Anticipated Turnover Scale
EE	Emotional Exhaustion
DP	Depersonalisation
PA	Personal Accomplishment
